# Expression of CLDN1 and CLDN10 in lung adenocarcinoma in situ and invasive lepidic predominant adenocarcinoma

**DOI:** 10.1186/1749-8090-8-95

**Published:** 2013-04-16

**Authors:** Zhenfa Zhang, Anlei Wang, Bingsheng Sun, Zhongli Zhan, Kexin Chen, Changli Wang

**Affiliations:** 1 Department of Lung Cancer, Lung Cancer Center, Tian Jin Medical University Cancer Institute & Hospital, Tian Jin, 300060, PR. China; 2 Department of Pathology, Tian Jin Medical University Cancer Institute & Hospital, Tian Jin, People's Republic of China; 3 Department of Epidemiology, Tian Jin Medical University Cancer Institute & Hospital, Tian Jin, People's Republic of China

**Keywords:** Lung adenocarcinoma in situ, Invasive lepidic predominant adenocarcinoma, Claudin 1, Claudin10

## Abstract

**Background:**

Non-mucinous bronchioloalveolar carcinoma (BAC) is considered the early stage of lung adenocarcinoma and is classified as the lung adenocarcioma in situ (AIS) by the International Association for the Study of Lung Cancer/American Thoracic Society/European Respiratory Society. This study was designed to investigate the gene expression differences between AIS (formerly non–mucinous BAC) and invasive lepidic predominant adenocarcinoma (LPA, formerly non-mucinous BAC pattern with >5 mm invasion, mixed type adenocarcinoma with BAC features) and to investigate the mechanism of the progression of lung adenocarcinoma in situ to invasive adenocarcinoma.

**Methods:**

Gene expression analysis was performed by using Agilent 4 × 44 K Whole Human Genome Oligo Microarray on 10 fresh frozen tissue samples of AIS and LPA, respectively. Real time RT-PCR was used to validate the differential expression of 13 genes selected by cDNA microarray on fresh frozen tissue samples from 41 patients with lung adenocarcinoma and 4 genes were confirmed. These 4 genes were then validated by western blotting. Immunohistochemical staining for these validated genes was performed on formalin-fixed, paraffin-embedded tissue samples from 81 cases of lung adenocarcinomna.

**Results:**

We identified a 13 gene expression signature by comparative analysis of gene expression. Expression of these genes strongly differed between AIS and LPA. Four genes (MMP-2, c-fos, claudin 1 (CLDN1) and claudin 10(CLDN10)) were correlated with the results of microarray and real time RT-PCR analyses for the gene-expression data in samples from 41 patients with lung adenocarcinoma. As confirmed by western blotting, the expression levels of MMP-2 and c-fos were higher in LPA than those in AIS; the expression levels of CLDN1 and CLDN10 in LPA were lower than those in AIS. Immunohistochemical staining for these genes in samples from 81 cases of lung adenocarcinoma demonstrated the expressions of CLDN1 and CLDN10 were correlated with overall survival of patients with lung adenocarcinoma.

**Conclusions:**

CLDN1 and CLDN10 may play important roles in the development of AIS to LPA. Overexpression of CLDN1 and CLDN10 indicates a favorable prognosis for overall survival in some patients with lung adenocarcinoma. Expression of CLDN10 may be regulated by the c-fos pathway.

## Background

Liebow first coined the term bronchioloalveolar carcinoma (BAC) in 1960, describing the tumor as a well-differentiated adenocarcinoma with neoplastic cells spreading along alveoli with little stromal reaction, no invasion, and preservation of alveolar architecture [1]. Based on the 2004 WHO classification guidelines [2], BAC has a distinct histological pattern of tumor cells growing along preexisting alveolar framework without evidence of stromal, pleural, or vascular invasion. However, a new international multidisciplinary classification was sponsored by the International Association for the Study of Lung Cancer, American Thoracic Society, and European Respiratory Society and non-mucinous BAC (≤3 cm) is classified as adenocarcinoma in situ (AIS) [3]. Some authors suggested that atypical adenomatous hyperplasia (AAH), adenocarcinoma in situ (formerly pure BAC), Minimally invasive adenocarcinoma (formerly BAC with focal invasion), invasive lepidic predominant adenocarcinoma (LPA, formerly adenocarcinoma with BAC features, invasive adenocarcinoma) might represent the developmental sequence of bronchioloalveolar stem cells rather than a classification system [4,5]. Stepwise progression from AAH through AIS to LPA was thus proposed. We made a hypothesis that there are some abnormal genes expressed in the progression from AIS to LPA, and these genes may be related with the poor prognosis of some patients with lung adenocarcinoma. In this study, we used gene microarrays to explore the differentially expressed genes between AIS and LPA. To our knowledge, it was the first study to investigate the roles of genes in the development of AIS to LPA.

## Methods

The lung cancer tissues analyzed in cDNA microarray study were obtained from the collection of fresh frozen lung cancer tissues in the tissue bank of Cancer Institute and Hospital, Tian Jin Medical University, Tian Jin, P.R. China, collected between 2000 and 2005. For the purpose of this study, 10 AIS and 10 LPA tissue samples were evaluated. The ethics committee of the university approved this study, and all patients participating in the study consented to sampling. Only tumor samples with neoplasticity of greater than 85% were included in the analysis. For each case, the histology slides were reviewed independently by two pathologists and tumors were classified according to the new IASLC classification system [3]. Clinical characteristics of the samples were provided in Table 1.

**Table 1:** Clinical features of AIS and LPA in the cohort1 and 2

**Factors**	**c1**		**c2**	
	**AIS**	**LPA**	**AIS**	**LPA**
Sex				
Male	7	2	5	13
Female	3	8	11	12
Age				
≤60	3	3	7	4
>60	7	7	9	21
Smoking				
No	9	8	16	12
Yes	1	2	0	13
Location				
Peripheral	10	8	16	19
Central	0	2	0	6
Surgical procedure				
Lobectomy	10	10	16	24
Pneumonectomy	0	0	0	1
laterality				
Left	5	4	10	9
Right	5	6	6	16
p N status				
N0	10	10	16	16
N1	0	0	0	6
N2	0	0	0	3
pT status				
T1	10	8	16	15
T2-3	0	2	0	10

c1: Clinical-pathological characteristics of the 10 patients with AIS and LPA used for cDNA microarray analysis.

c2: Clinical-pathological characteristics of the 41 patients in the validation cohort.

In order to validate the selected genes, we studied frozen specimens of lung-cancer tissues from 41 randomly selected patients with lung adenocarcinoma who underwent surgical resection at the Tianjin Medical University Cancer Institute & Hospital. None of the patients received the neoadjuvant chemotherapy (Table 1).

81 paraffin-embedded tissues of lepidic predominant adenocarcinoma (LPA) were obtained from Tianjin Cancer Institute & Hospital, Tianjin Medical University, Tianjin, China. Patient medical records were retrospectively reviewed to assess clinical characteristics and survival. There were 30 (37.0%) males and 51 (63.0%) females with ages ranging from 32 to 78 years, and a median age of 61 years. Those who had smoked <100 cigarettes in their lifetimes were defined as never smokers as traditionally used in epidemiological studies, while the remaining cases were considered as smokers. The routine preoperative workup included contrast CT scans of the chest, whole body bone scanning, liver sonography, flexible bronchoscopy, and brain magnetic resonance imaging. 16 patients underwent PET-CT scans. In our hospital, if a contrast CT or PET-CT showed no mediastinal lymph node enlargement, a cervical mediastinoscopy or EBUS/EUS-TBNA is not routinely performed. 9 patients in our study underwent mediastinoscopy. No neoadjuvant therapy was applied to any of the 81 patients. All these patients had systematic lymph node dissection or sampling performed. Prior consents from the patients and approval from the Research Ethics Committee of Tianjin Cancer Institute & Hospital of Tianjin Medical University were obtained. Histological classification of the samples was graded according to the WHO histologic classification. Clinicopathological variables were summarized in Table 2.

**Table 2:** Factors and prognosis of patients with LPA by Univariate Analysis

**Factors**	**n**	**5-year survival (%)**	**p**
Sex			
Male	30	54.73	0.7494
Female	51	46.43	
Smoking			
No	53	56.39	0.1286
yes	28	40.56	
Proportion of lepidic growth (BAC) in adenocarcinoma^*^			
≤50% (including 0%)	50	35.69	0.0375
>50%	31	58.32	
Age			
≤60 years	25	52.85	0.6028
>60 years	56	47.74	
Location			
Central	7	42.86	0.2521
Peripheral	74	50.47	
Staging			
I	50	69.87	0.0002
II	26	38.46	
III	5	15.63	
T stage			
T1	43	60.63	0.0142
≥T2	38	43.38	
N stage			
N0	62	63.20	0.0004
N1	16	38.46	
N2	3	15.63	
Surgical procedure			
Lobectomy	79	53.85	
Pneumonectomy	2	49.12	
Laterality			
Left	38	52.12	0.2873
Right	43	48.29	
ECOG performance status			
0	75	50.83	0.4872
1	6	49.15	
Histologic differentiation			
Well	58	60.37	0.0089
Moderate/poorly	23	41.28	
Claudin 1			
-	33	26.75	0.0034
+	48	63.20	
c-fos			
-	28	55.19	0.8438
+	53	29.43	
Claudin 10			
-	25	30.30	0.0019
+	56	59.75	
MMP-2			
-	19	12.00	0.3208
+	62	41.81	
Combination of CLDN1&CLDN10			
both factors expressed	35	68.90	0.0000
either factor expressed	35	44.32	
neither factor expressed	11	0	

^*^: We defined 50% as a cutoff for lepidic growth in these tumors because some studies showed that if the lepidic growth was more than 50%, the 5-year survival of patients with T1 lung adenocarcinoma was much better than those with less than 50% lepidic growth [6-9].

We recommended that patients have follow-up visits every 3 months for 2 years, then visits every 6 months for 3 years and then annual visits. All patients were followed up until death or the last day of follow-up (December 31, 2010). The median length of follow-up for surviving patients was 57 months (range, 9–101 months).

Follow-up information was obtained either from hospital case records, or a questionnaire completed by the chest physician or general practitioner via telephone or mail, or from death certificates. The follow-up protocol included physical examination of patients, tumor markers, chest radiography, CT scanning of the chest, liver sonography, and whole body bone scanning. Overall survival was measured from the date of operation until the date of death. Patients who died of other causes without evidence of recurrent disease or who were unavailable for follow-up were censored either at the time of death or at the last follow-up.

### Microarray analysis

RNAs from 10 AIS and 10 LPA were extracted with TRIZOL reagent (Invitrogen, MD, USA) and purified with RNeasy MINI Kit (Qiagen, CA, USA) according to the manufacturer^’^s instructions. All samples included for analysis were determined to be of high quality RNA (OD260/280 = 1.9–2.1. NANODROP 2000c, Gene Company Limited). Low RNA Input Fluorescent Linear Amplification Kits (Agilent Technologies) were used to generate fluorescently labeled cRNA. Briefly, MMLV-Reverse transcriptase and dT-T7 primer were used to amplify mRNA from total RNA samples into dsDNA. Cyanine 3-labeled cRNA was produced using T7 polymerase. The labeled cRNA was purified using RNeasy Mini kits and concentration was measured using a spectrophotometer. cRNA (875 ng) was fragmented for 30 min at 60°C and subsequently hybridized to 4x44K Whole Human Genome Oligo Microarray chip (Agilent Technologies) in stainless steel hybridization chambers. Hybridization was carried out over 17 h in the dark in a rotating hybridization chamber set at 60°C and 4 rpm. After hybridization, slides were washed in Gene Expression Wash Buffer 1 and 2 for 1 min and then dried by centrifugation at 2000 rpm for 2 min. Microarrays were scanned with a dynamic autofocus microarray scanner (Agilent Microarray Scanner- G2565AA, Agilent Technologies,) using Agilent-provided parameters (PMT was set at 100%, and scan resolution was set to 5 μm). The Agilent Feature extraction software (FE) (Agilent Technologies) was used to extract and analyze the signals. This software calculates log ratios and p-values for valid features on each array and provides a confidence measure of gene differential expression performing outlier removal and background subtraction. Furthermore, FE filters features that are not positive or significant with respect to background or are saturated. FE was also used to perform linear and LOWESS dye normalization to correct dye bias. The raw data and associated sample information were loaded and processed by GeneSpring® 10 (Agilent Technologies). Statistical analysis was performed using background-corrected mean signal intensities from each dye channel. Microarray data were normalized using intensity-dependent global normalization (LOWESS). Differentially expressed RNAs were identified using a filtering by the Benjamini and Hochberg False Discovery Rate (p < 0.05) to minimize selection of false positives. Of the significantly differentially expressed RNA, only those with greater than 5-fold increase or 5-fold decrease in expression compared to the controls were used for further analysis. We identified 353 up- and 187 down-regulated genes from Agilent 4x44K Whole Human Genome Oligo Microarray.

### Quantitative real-time reverse transcription-PCR

To validate the expression levels of genes found on microarray analysis, real-time RT-PCR was performed on 13 genes and the control gene GAPDH, with the use of specific TaqMan probes and primer sets (Table 3). The transcripts were amplified with reagent (TaqMan One-Step RT-PCR Master Mix Reagent, Applied Biosystems) and a sequence detection system (ABI Prism 7900HT, Applied Biosystems). Gene expression was quantified in relation to the expression of GAPDH using sequence detector software with the relative quantification method (Applied Biosystems).

**Table 3:** The primers of 13 genes

**Gene**		**Primer (5**^**′**^**-3**^**′**^**)**	**Genbank**	**Product size(bp)**
CLDN7	Forward	GGAGTGTCTAGATGCCTGAAA	NM_001185022.1	118
	Reverse	CCCAACCCAAGAGGACTATAC		
SMURF2	Forward	AGAGTGCCCAGGGATCTTA	NM_022739	91
	Reverse	CTCTGCCTGTTGCCGTATTA		
CLDN1	Forward	GATAGCAATCTTTGTGGCCACCGT	NM_021101.4	205
	Reverse	TTCGTACCTGGCATTGACTGGG		
C-FOS	Forward	CTTCCTGTTCCCAGCATCAT	NM_005252.3	89
	Reverse	CTGCATAGAAGGACCCAGATAG		
MET	Forward	AAATGTGTCGCTCCGTATCC	NM_001127500.1	99
	Reverse	GCACTATGATGTCTCCCAGAAG		
VCAM1	Forward	AATGCCCATCTATGTCCCTTG	NM_001199834.1	98
	Reverse	ACTCCTCCAGTTCTCTCATCTT		
WIF1	Forward	TAGAGGGAGAGCAGTGTGAA	NM_007191.4	103
	Reverse	CCCTGGTAACCTTTGGAACA		
FGFR1	Forward	GGAGTTAATACCACCGACAAAGA	NM_001174067	110
	Reverse	TGGGAGAGTCCGATAGAGTTAC		
WNT2	Forward	GCACCTAAAGCCTACCCTATTC	NM_003391.2	92
	Reverse	GCCTGTCATGCTATTTCCAAAG		
MMP2	Forward	TGCTGAAGGACACACTAAAGAA	NM_001127891.1	88
	Reverse	CGCATGGTCTCGATGGTATT		
SOCS1	Forward	CTTCTGTAGGATGGTAGCACAC	NM_003745.1	98
	Reverse	AGGAAGAGGAGGAAGGTTCT		
MUC4	Forward	CTAAAGGTCACGTGGGTCAA	NM_004532.5	120
	Reverse	CTGGTAGAGAAACAGGGCATAG		
SP1	Forward	GATGTGTGGGCTTCTGAGTTTA	NM_001251825.1	100
	Reverse	ACTGGCTGATGCTCCTTATTG		

### Western blotting

In brief, 100 mg frozen tissue was homogenized, and extracts were centrifuged at 10,000 g for 5 minutes at 4°C to remove debris. Then, 10 uL supernatant was heated in a boiling water bath for 4 minutes and subjected to sodium dodecyl sulfate (SDS)-polyacrylamide gel electrophoresis. The electrotransfer of proteins from the gel to a nitrocellulose membrane was performed for 30 minutes using a semidry transfer system (BioRad, Hercules, CA). The membrane was then soaked in 5% defatted dry milk diluted with phosphate-buffered saline containing 0.02% Tween20 to reduce nonspecific protein binding. Next, the membrane was incubated with 50 uL antibody (MMP-2, c-fos: Santa Cruz Biotechnology, Inc, Santa Cruz, CA; CLDN1, CLDN10: Zymed Laboratories Inc, San Francisco, CA) for 45 minutes at room temperature and then treated with a suitable second antibody for 30 minutes at room temperature. Enhanced chemiluminescence analysis was performed according to the manufacturer’s instructions, and the image was developed in a film in a darkroom.

### Immunohistochemistry

Paraffin sections (4 μm) from samples were deparaffinized in 100% xylene and re-hydrated in descending ethanol dilutions according to standard protocols. Heat-induced antigen retrieval was performed in EDTA buffer (pH 9.0) for 3 min at 100°C. Endogenous peroxidase activity and non-specific antigens were blocked with peroxidase blocking reagent containing 3% hydrogen peroxide and serum, followed by incubation with each antibody overnight at 4°C. After washing, the sections were incubated with biotin-labeled secondary antibody for 40 min at 37°C, and were subsequently incubated with streptavidin-conjugated horseradish peroxidase (HRP). The peroxidase reaction was developed using 3, 3-diaminobenzidine chromogen solution in DAB buffer substrate (ChemMate TM EnVision TM Detection Kit, Dako, USA). Sections were visualized with DAB counterstained with hematoxylin, mounted in neutral gum, and analyzed using a bright field microscope [10]. We counted 800 of the cancer cells. All immunostained sections were examined by at least two pathologists. Positive staining was defined as the presence of immunoreactivity in at least 10% of cancer cells. We defined the 10% immunoreactivity of the immunostained sections as the cut-off point arbitrarily.

### Data analysis

The average intensity for each gene in the microarray was assessed. To reduce variation in microarray analysis, the intensity values for samples in each microarray were rescaled by means of a quantile normalization method [11]. Each intensity value was then log-transformed to a base-2 scale. Finally, the gene expression intensity values were transformed to ordinal coding values, according to the ranking of the level of gene expression among the 550 genes in 41 patients. The intensity value was coded as 1 for expression levels ranked at or below the 25th percentile of the total gene expression, 2 for levels above the 25th and at or below the 50th percentiles, 3 for levels above the 50th and at or below the 75th percentiles, and 4 for levels above the 75th percentile. We used univariate Cox proportional-hazards regression analysis to evaluate the association between survival and the level of expression of each gene from microarray analysis. For genes that were significantly correlated with survival, we performed real-time PCR for further analysis [12].

The *x*^2^ test was used to evaluate for associations between c-fos/MMP2 and CLDN1/CLDN10 expression. Survival rates were calculated by the Kaplan-Meier method. Comparisons were made with log-rank test, and the Cox proportional hazards ratio model was used to investigate the simultaneous effect of multiple predictors on survival. All tests of significance were two-sided, and differences were considered statistically significant at *p* values less than 0.05. The SPSS software (version 10.0; SPSS, Chicago, Illinois) was used for the analysis.

## Results

### 1. c-fos, MMP-2,CLDN1 and CLDN10 were differently expressed between AIS and LPA

**Figure 1:**
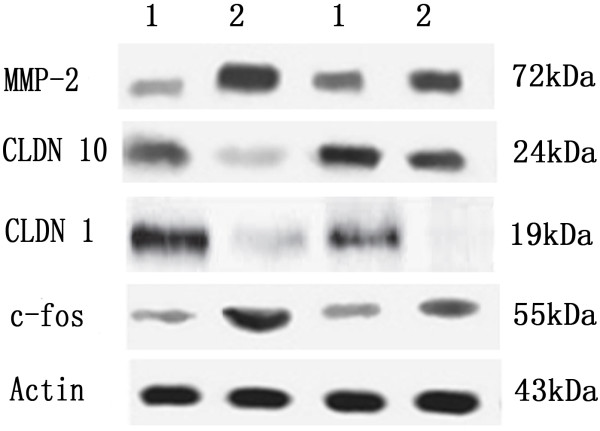
**Expression of c-fos, MMP-2, claudin 1 and claudin 10 in AIS and LPA by Western blotting.** The blots were subsequently probed with anti-actin antibody as a loading control. 1: AIS: adenocarcinoma in situ 2: LPA: lepidic predominant adenocarcinoma.

To investigate the molecular factors associated with lung adenocarcinoma development, we investigated gene expression changes between AIS and LPA. For this study, 10 AIS and 10 LPA tissues were selected. The clinicopathologic data were summarized in Table 1. Using stringent selection criteria (fold-change ≥5 and p-value < 0.001), we identified 353 up- and 187 down-regulated genes from Agilent 4x44K Whole Human Genome Oligo Microarray (data not shown). The univariate Cox regression analysis showed that the expression levels of 13 genes correlated with disease-free survival of patients with lung adenocarcinoma (Table 4). All 13 differentially expressed genes were chosen for RT-PCR validation. There was a significant correlation between the results of microarray and RT-PCR analyses for the gene expression data for 4 of the 13 genes in samples from 41NSCLC patients (Table 4). These four genes including c-fos, MMP-2, CLDN1 and CLDN10 were validated by western blotting (Figure 1). Both up- and down- regulated genes were identified. The expression levels of C-fos and MMP-2 were higher in LPA disease than those in AIS; While the expression levels of CLDN1 and CLDN10 were lower in LPA.

**Table 4:** Validation of the 13 differentially expressed genes in 41 patients with AIS LPA

**Gene name**	**Fold regulation**	**Up/down**	**P**^**※**^	**HR**	**Correlation coefficient**^**▲**^	P^*^
claudin 7 (CLDN7)	0.0691	Down	<0.01	0.432	0.68	<0.01
SMAD specific E3 ubiquitin-- protein ligase2 (SMURF2)	5.8528	Up	<0.01	2.422	0.58	<0.01
claudin 1 (CLDN1)	0.0344	Down	0.001	1.731	0.44	<0.01
FBJ murine osteosarcoma viral oncogene homolog (c-fos)	7.8182	Up	0.02	0.75	0.64	<0.01
met-proto-oncogene (MET)	5.5674	Up	<0.01	0.455	0.26	0.21
Vascular cell adhesion molecule 1 (VCAM1)	0.0593	Down	<0.01	2.156	0.25	0.23
WNT inhibitory factor 1 (WIF1)	0.1727	Down	<0.01	1.727	0.20	0.45
Fibroblast growth factor receptor 1 (FGFR1)	8.3246	Up	0.001	0.611	0.14	0.66
Wingless-typeMMTVintegrationsitefamilymember 2 (wnt 2)	6.1408	Up	<0.01	0.497	0.18	0.68
Matrix metallopeptidase 2 (MMP-2)	9.6869	Up	0.038	1.321	−0.19	0.59
Suppressor of cytokinesignaling1 (SOCS1)	5.0212	Up	0.001	1.671	0.05	0.78
Mucin 4, cell surface associated (MUC4)	0.1278	Down	0.012	0.717	0.02	0.82
Sp1 transcription factor (sp1)	0.1567	Down	0.049	0.793	0.01	0.89

P^※^ values were estimated by univariate Cox regression analysis of the microarray data.

^▲^: Correlation Coefficient for Microarray Results vs. Real-Time RT-PCR Results.

P^*^values for the correlation coefficients were estimated by Spearman’s rank-correlation test.

### Immunohistochemical analysis

**Figure 2:**
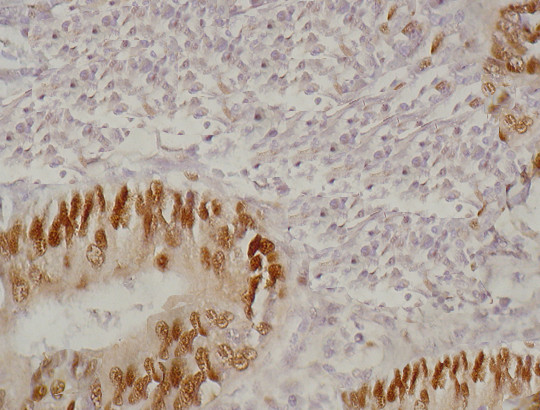
**Immunohistochemical staining of c-fos expression in the lung adenocarcinoma tissues.** c-fos showed nuclear staining pattern.

**Figure 3:**
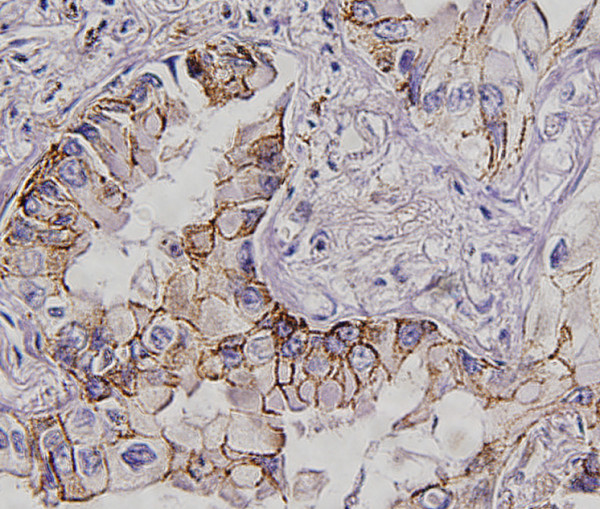
**Immunohistochemical staining of CLDN1 expression in the lung adenocarcinoma tissues.** CLDN1 was expressed in the cell membrane.

**Figure 4:**
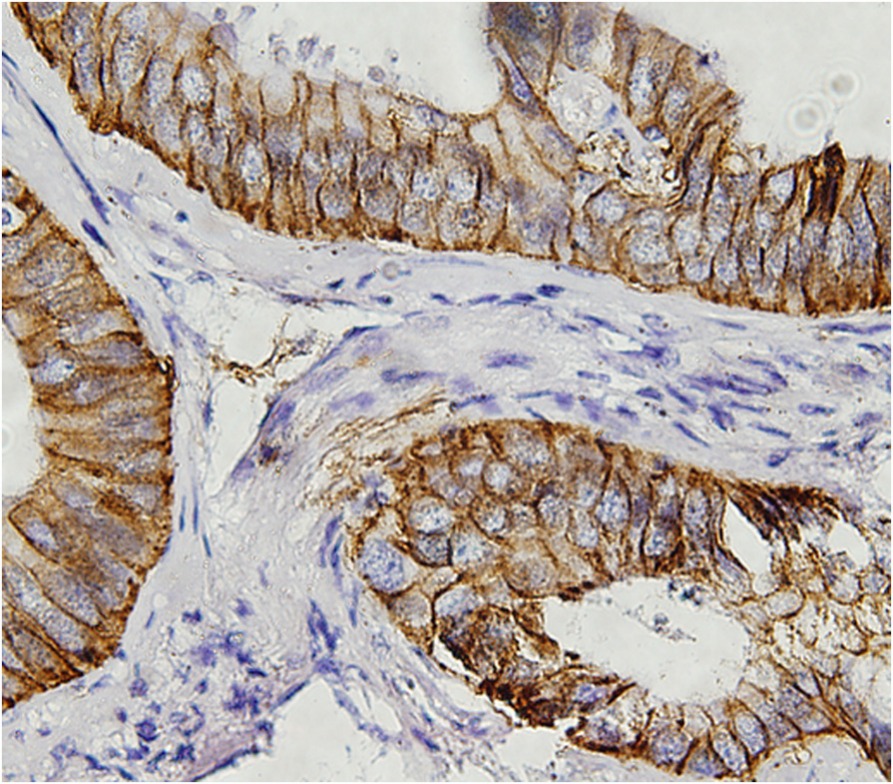
**Immunohistochemical staining of CLDN10 expression in the lung adenocarcinoma tissues.** CLDN10 showed membrane staining pattern.

**Figure 5:**
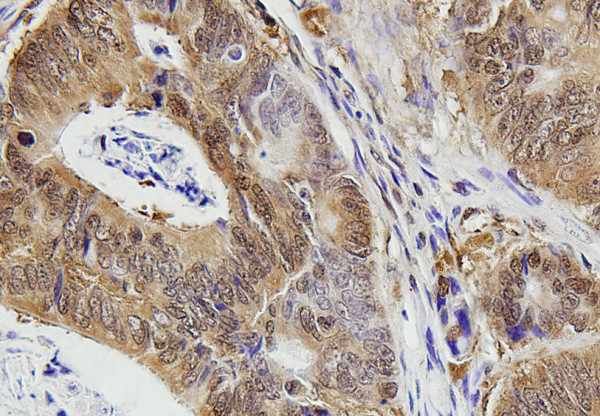
**Immunohistochemical staining of MMP-2 expression in the lung adenocarcinoma tissues.** MMP-2 was expressed in the cell membrane, cytoplasm and extracellular space.

In order to know whether the expression levels of these 4 genes had a prognostic value in lung adenocarcinoma, we obtained the paraffin-embedded tissue blocks from 81 consecutive patients with adenocarcinonma for immunohistochemical staining. The expression of c-fos in most of the cancer cells showed nuclear staining pattern (Figure 2) and the nuclear positive rate was 62.2% (53/81). The CLDN1 (Figure 3) and CLDN10 (Figure 4) showed the membrane staining pattern, and the positive rate was 59.3% (48/81) and 69.1% (56/81), respectively. The MMP-2 showed membrane, cytoplasm and extracellular space staining (Figure 5), and the positive rate was 76.5% (62/81). The expression of claudin 10 was inversely related with the expression of c-fos (Table 5).

**Table 5:** The expressions of claudin1, claudin 10, c-fos and MMP-2 in lung adenocarcinoma

	**Claudin1**		**p**	**Claudin 10**		**p**
	**+**	**−**		**+**	**−**	
c-fos						
+	28	25	0.105	30	23	0.001
-	20	8		26	2	
MMP-2						
+	38	24	0.502	44	18	0.519
-	10	9		12	7	

### Predicting prognosis

**Figure 6:**
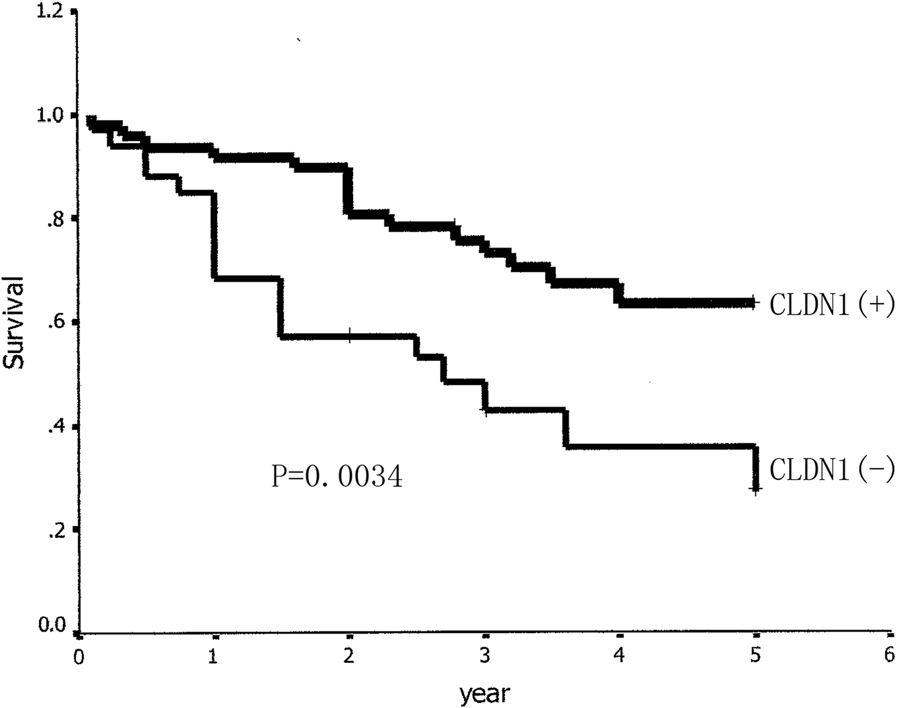
**Relationship between expression of CLDN1 and survival of patients with lung adenocarcioma.** Patients with positive CLDN1 expression had a better survival than those with negative expression (P = 0.0034).

**Figure 7:**
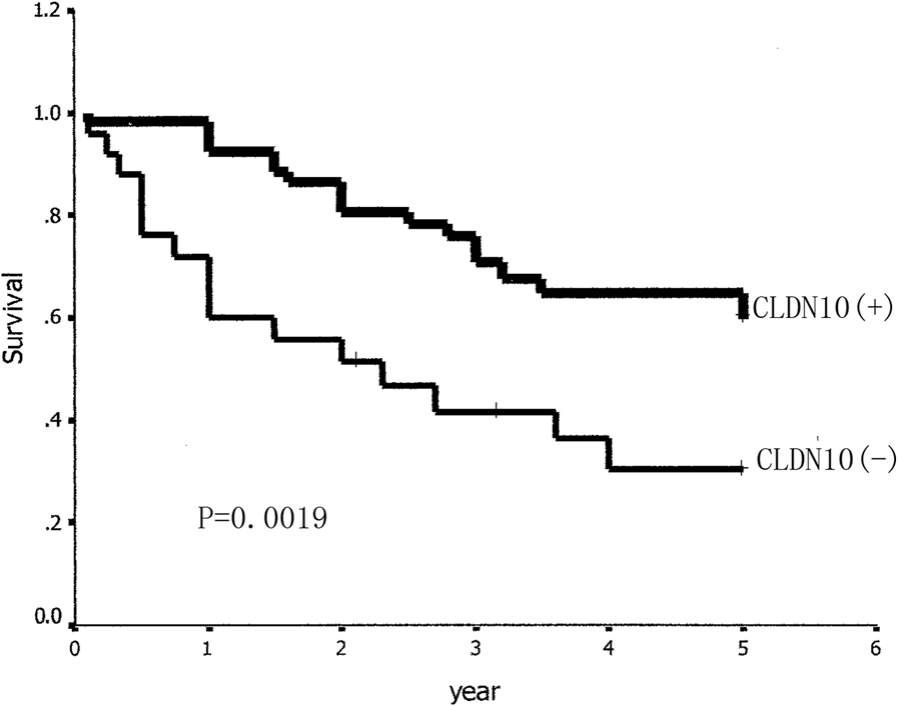
**Relationship between expression of CLDN10 and survival of patients with lung adenocarcioma.** Patients with positive CLDN10 expression had a better survival than those with negative expression (P = 0.0019).

**Figure 8:**
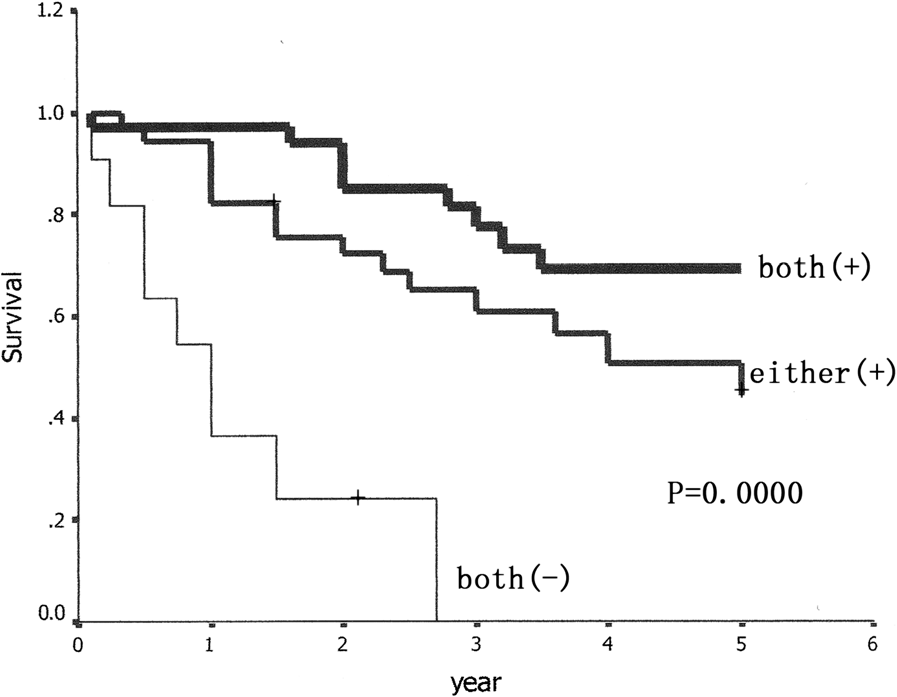
**Patients with the expression of both CLDN 1 and CLDN10 had a better 5-year overall survival than those with either and neither expression of the two factors (P = 0.0000).** Both (+): Both CLDN 1 and CLDN10 were positively expressed. Either (+): Either CLDN 1 or CLDN10 was positively expressed. Both (−): Neither of the two factors was positively expressed.

Univariate analysis revealed that staging, the proportion of lepidic growth (BAC) in adenocarcionma, lymph node involvement, the expression of CLDN 1, CLDN 10 and T stage (*p* = 0.0002, *p* = 0.0375, *p* = 0.0004, *p* = 0.0034, *p* = 0.0019, *p* = 0.0142, respectively; Table 2) were prognostic factors. Patients with positive expression of CLDN 1/CLDN 10 had a survival significantly longer than those with negative expression of these two factors (Figures 6 and 7). We made a survival analysis of the combination of CLDN 1 and CLDN 10, and found that patients with positive expression of these two factors had a better 5-year overall survival than those with either or neither expression of the factors (P = 0.0000, Figure 8). Table 6 summarizes the multivariate analysis of prognostic value of prognostic factors, which were determined by univariate analysis (*p* <0.05), in overall survival in 81 patients. In this study, staging (*p* = 0.004), lymph node involvement (*p* = 0.024), T stage (*p* =0.041), CLDN 1 (*p* =0.038), and CLDN 10 (*p* =0.029) were found to be related with overall survival of patients with LPA.

**Table 6:** Factors and prognosis of patients with LPA by Cox proportional hazards ratio model

**Factors**	**OR**	**95% CI**	**p**
Proportion of lepidic growth (BAC) in adenocarcinoma			
≤50%	0.513	0.256-0.834	0.098
>50%			
Staging			
I	0.334	0.251-0.635	0.004
II			
III			
N stage			
N0	0.245	0.011-0.085	0.024
N1			
N2			
T stage			
T1	0.587	0.015-0.089	0.041
T2-3			
Histologic differentiation			
Well	0.382	0.009-0.765	0.035
Moderate/poorly			
Claudin 1			
+	0.415	0.176-0.767	0.038
-			
Claudin10			
+	0.352	0.165-0.867	0.029
-			

CI: confidence interval; OR:odds ratio.

## Discussion

Literatures regarding the status of claudins in various cancers are constantly expanding, and the expression levels of claudins seem to change in a tissue specific manner. Tan et al. [13] showed that the expression and distribution of claudin-1were associated with cell dissociation status in pancreatic cancer cells through mitogen-activated protein kinase 2 activations. Claudin-7 was found to be decreased in invasive ductal carcinomas [14], head and neck cancer [15] and metastatic breast cancer [16]. Claudin-3 and −4 were frequently elevated in various cancers including pancreatic ductal adenocarcinoma [17], prostate, uterine, ovarian cancer [18], while hepatocellular and renal carcinomas expressed lower levels of claudins-4 and −5 [19]. While, lower expression of claudin-2 was also seen in breast and prostatic carcinomas, expressions of claudin1 and claudin7 that were undetectable in normal cervical squamous epithelium increased in the cervical neoplasia [19,20]. Recent studies have shown that the expression of certain claudins especially claudin-1 and claudin-4 increases during metastasis and genetic inhibition of their expression has profound effect on the metastatic abilities of cancer cells though in a tissue specific fashion [21-24]. mRNA and protein expressions of claudin-1 were found to have prognostic values in lung adenocarcioma [22]. Our study found that the expressions of CLDN1 and CLDN10 were higher in AIS than those in LPA. The Cox proportional hazard models indicated that claudin 1 and claudin 10 were prognostic predicators. Our results suggested that claudin 1 and claudin 10 may play important roles in the progression of AIS to invasive lung adenocarcinoma.

Decreased expressions of claudin 1 and claudin 10 might lead to the compromised tight Junctions’ function and, thus, neoplasia progression was easy to comprehend. Furthermore, it is postulated that claudins may also affect cell signaling pathways. Cell-cell adhesion proteins are known to play important role in cellular transformation when displaced from their normal membrane localization and can serve as oncogenic molecule. The best studied molecule was *β*- catenin, which could serve as a cell-cell adhesion molecule when expressed in its normal cellular localization [25]. Expression of specific claudin family members could be regulated by Wnt signaling pathway. Claudin-1 and claudin-2 were shown to be target genes regulated by *β*-catenin signaling [26,27]. In our study, we found the expression of claudin 10 was inversely related with the expression of c-fos. c*-*Fos, a well-established oncogene, is considered to play a critical role in tumorigenesis, proliferation and transformation, angiogenesis, tumor invasion, and metastasis, and its expression is associated with poor clinical outcomes, even though some studies suggested that c-Fos might also have a proapoptotic role [28,29]. Our study suggested that the expression of claudin 1(p = 0.105) and claudin 10(p = 0.027) may fuction as cancer cell invasion and metastatic suppressor in lung adenocarcinoma through c-fos signal pathway.

Our study also found a higher expression of MMP-2 in LPA, suggesting the role of MMP-2 in the development of lung adenocarcinoma. Matrix metalloproteinases (MMP) are a family of over 20 extracellular, zinc-dependent proteolytic enzymes capable of degrading multiple components of the extracellular matrix. These enzymes play important roles not only in normal physiologic conditions, such as matrix homeostasis, but also in pathologic processes, including tumor progression where their expression is associated with invasive and metastatic behavior [30,31]. Not surprisingly, our study indicated that MMP-2 may play an important role in the progression of lung adenocarcinoma.

Our study had some shortcomings: first, the number of the patient samples was small. We just randomly selected some patients for validation who had LPA and full follow-up information, so the study may have the selection bias. Second, the cDNA microarray showed many abnormally expressed genes between AIS and LPA disease. We only selected 13 genes by our way of statistical analysis, and after validation, only 2 genes were proved to have clinical significance. We have to admit that there are some other genes that may have some clinical value, even the other putative oncogenes and suppressor genes in the 13 differentially expressed genes in our study are likely to have a role. But the genes we selected were proved to have some clinical significance in lung adenocarcinoma. We believe that the progress, invasion and metastasis of lung adenocarcinoma are so complicated that they cannot be explained clearly by a simple study. Our results only provide a way to know the mechanism of lung cancer cell developing and to predict prognosis of lung adenocarcinoma, although it remains to be determined through further studies.

## Conclusions

Our study shows that CLDN1 and CLDN10 may play important roles in the development of AIS to LPA. Overexpression of CLDN1 and CLDN10 indicates a favorable prognosis for overall survival in some patients with lung adenocarcinoma. Cell-cell adhesion proteins play important roles in cellular transformation. Decreased claudin 1 and claudin 10 expression may lead to the compromised tight Junctions’ function and the neoplasia progression. Furthermore, it is postulated that claudins may also affect cell signaling pathways. Expression of CLDN10 may be regulated by the c-fos pathway, which is related with lung cancer progression.

## Abbreviations

AIS: Lung adenocarcioma in situ; BAC: Bronchioloalveolar carcinoma; LPA: Lepidic predominant adenocarcinoma; CLDN1: Claudin1; CLDN10: Claudin10

## Competing interests

The authors declare that they have no competing interests.

## Authors’ contributions

ZZ participated in its design and helped to draft the manuscript. AW carried out the experiments and drafts the manuscript. BS participated in the sequence alignment, participated in its design and coordination and helped to draft the manuscript. ZZ participated in the pathologic review. KC performed the statistical analysis. CW participated in its design and coordination. All authors read and approved the final manuscript.
